# AI-based synthetic CT attenuation correction enables reliable quantitative SPECT in unilateral condylar hyperplasia

**DOI:** 10.1186/s40658-026-00879-z

**Published:** 2026-04-20

**Authors:** Anna Rebeka Kovács, Enikő Fanni Juhász, Péter Czina, Ádám Budai, József Varga, Borbála Husztik, Ákos Kovács, Melinda Szoliková, Róbert Boda, Kincső Sára Kovács, Ildikó Garai, Sándor Kristóf Barna

**Affiliations:** 1 Division of Nuclear Medicine and Translational Imaging, Department of Medical Imaging, Faculty of Medicine, University of Debrecen, Debrecen, 4032 Hungary; 2 Jávorszky Ödön Hospital, Vác, 2600 Hungary; 3 Scanomed Nuclear Medicine Center, Debrecen, 4032 Hungary; 4 Mediso Ltd, Budapest, 1037 Hungary; 5 Department of Oral and Maxillofacial Surgery, Faculty of Dentistry, University of Debrecen, Debrecen, 4032 Hungary; 6 Division of Radiology and Imaging Science, Department of Medical Imaging, University of Debrecen, Debrecen, 4032 Hungary

**Keywords:** Artificial intelligence, Attenuation correction, Quantitative SPECT, Synthetic CT, Unilateral condylar hyperplasia

## Abstract

**Purpose:**

Unilateral condylar hyperplasia (UCH) is a rare mandibular growth disorder in which accurate assessment of condylar metabolic activity is essential for surgical decision-making. Quantitative ^99m^Tc-methylene diphosphonate (MDP) SPECT/CT is commonly used for this purpose; however, CT-based attenuation correction (CTAC) increases radiation exposure and may be affected by registration errors. Artificial intelligence (AI)-generated synthetic CT (SyCT) has been proposed as a CT-independent alternative. This study aimed to evaluate the agreement between SyCT-based attenuation correction (SyCTAC) and conventional CTAC in quantitative SPECT imaging of UCH.

**Methods:**

This retrospective study included 14 patients with UCH who underwent ^99m^Tc-MDP SPECT/CT. SPECT images were reconstructed with identical parameters using either CTAC or AI-generated SyCTAC. Spherical volumes of interest were placed over both mandibular condyles and the clivus, and maximum and mean standardized uptake values were measured. Relative uptake fractions normalized to the summed condylar activity were calculated for the affected side, while condylar uptake normalized to the clivus was evaluated for both sides. Agreement between CTAC- and SyCTAC-derived indices was assessed using Bland–Altman analysis and linear mixed-effects models.

**Results:**

Visual evaluation revealed no relevant differences in image quality between the two reconstructions. Relative uptake fractions normalized to the summed condylar activity showed good agreement between methods. Clivus-normalized ratios demonstrated a small positive bias.

**Conclusion:**

AI-generated SyCT provides attenuation correction comparable to CTAC for clinically relevant relative condylar uptake assessment in UCH, supporting its use as a low-radiation alternative for quantitative mandibular SPECT imaging.

## Introduction

Unilateral condylar hyperplasia (UCH) is a rare mandibular growth disorder characterized by progressive enlargement of one condylar process, leading to facial asymmetry, malocclusion and temporomandibular joint (TMJ) dysfunction [1–3]. The condition may manifest at any age but most commonly arises during early adolescence and shows a higher prevalence in women [2, 3]. Although multiple etiological mechanisms have been proposed—including aberrant endochondral ossification, localized metabolic dysregulation, vascular abnormalities, trauma, infection, genetic factors, and hormonal influences—the precise pathogenesis of UCH remains unclear [1, 3].

The clinical importance of differentiating active from inactive forms of the disease is substantial, as therapeutic strategies vary markedly according to the growth status of the affected condyle [4]. In cases of active UCH, condylectomy is commonly indicated to prevent further progression of asymmetry, whereas orthognathic surgery alone may be suitable for inactive disease. Accurate assessment of condylar growth activity is therefore critical for determining both the optimal timing and the appropriate type of surgical intervention [2, 4].

Bone scintigraphy, particularly single-photon emission computed tomography (SPECT) using ^99m^Technetium (^99m^ Tc)-labeled diphosphonates, has been widely used in the evaluation of UCH because radiotracer uptake reflects osteoblastic activity and, by implication, active condylar growth [1, 5]. In clinical practice, several semi-quantitative parameters can be used to assess condylar growth activity, including the condylar relative uptake, the condyle-to-L4 vertebral uptake ratio, the condyle-to-clivus uptake ratio, and the condyle-to-external-standard ratio, all of which assist in identifying active condylar hyperplasia [1]. The condylar relative uptake, defined as the ratio of the unilateral count to the total count of both condyles, is the most commonly used parameter for evaluating condylar activity. A condylar relative uptake of 55% or greater—corresponding to an intercondylar difference of at least 10%—is generally considered indicative of active UCH in the literature [1, 4, 5].

Quantitative SPECT, particularly through the use of standardized uptake values (SUVs), increases the objectivity of metabolic assessments, potentially reducing interobserver variability [1, 4, 6] and improving the robustness of surgical planning. A major limitation of SPECT is photon attenuation, a physical phenomenon in which gamma rays traversing the body are absorbed or scattered by tissues of varying density, leading to non-uniformities in the detected signal. These effects can substantially degrade diagnostic accuracy. Therefore, attenuation correction (AC) is required to compensate for photon loss and to improve the fidelity of the reconstructed images [7].

Reliable quantitative SPECT imaging depends critically on accurate AC. Conventional computed tomography (CT)-based attenuation correction (CTAC) is the standard method for generating attenuation maps [4, 6], but it is associated with several limitations. CT acquisition contributes additional radiation dose [8, 9], which is particularly relevant in UCH, as the condition often affects young patients who may require repeated imaging during follow-up [2]. In this population, adherence to the ALARA (As Low As Reasonably Achievable) principle is of paramount importance, necessitating the reduction of unnecessary radiation whenever possible without compromising diagnostic value [10]. Furthermore, CT-corrected data may be compromised by motion artifacts or by misregistration between the SPECT and CT volumes, both of which can adversely affect quantification [7], especially in anatomically small regions such as the TMJ.

These limitations have motivated growing interest in alternative AC strategies that do not require CT acquisition. Artificial intelligence (AI), particularly deep learning technologies, has recently demonstrated broad applicability in medical imaging. Networks like convolutional neural network (CNN) or generative adversarial network (GAN) can create CT-equivalent attenuation maps (µ-maps or synthetic CT [SyCT]) directly from emission data without requiring a CT scan [7, 11, 12].

Given the increasing interest in CT-independent quantification and the high clinical value of reliable condylar uptake assessment in UCH, it is essential to determine whether AI-generated SyCT-based attenuation correction (SyCTAC) can reliably reproduce CTAC-derived uptake measurements. Therefore, the aim of this study was to evaluate the agreement between SyCTAC and CTAC in quantifying relative condylar uptake on ^99m^Tc-methylene diphosphonate (MDP) SPECT/CT in patients with UCH. The goal was to determine whether SyCTAC can serve as a viable alternative to conventional CTAC for quantitative assessment of mandibular condylar activity.

## Materials and methods

### Study population

In this study, with the permission of the Ethics Committee of the Hungarian Medical Research Council (ETT-TUKEB), we retrospectively analyzed the scans of 14 patients who underwent ^99m^Tc-MDP SPECT/CT examinations for UCH at Scanomed Ltd. between 2014 and 2024. The patients’ mean age was 24.29 years, with a median age of 20.46 years. The oldest patient was 42.29 years old, while the youngest was 16.29 years old. There were three patients under the age of 18.

### Bone SPECT/CT imaging

The mandibular bone SPECT procedure was carried out 2 h after the intravenous administration of a dose of 600 MBq ^99m^Tc-MDP for patients over 18 year and normalized according to the EANM (European Association of Nuclear Medicine) Pediatric Dosage Card for patients under 18 [13].

The SPECT images were obtained using a triple-headed AnyScan^®^ TRIO SPECT/CT (Mediso Ltd., Budapest, Hungary) with low energy high resolution high sensitivity (LEHR-HS) collimators and the following parameters: frame size: 128 × 128 matrix, frame time: 15 s, 120 views and the spectrum energy center was 141 keV with 20% window. CT images were acquired in the same AnyScan^®^ TRIO SPECT/CT equipment without contrast enhancement, applying the following parameters: slice thickness: 2.5 mm, pitch: 1.5, 90/min rotation speed, resolution: 512 × 512, energy: 120 kV. Automatic dose reduction was enabled, utilizing the following parameters: mAs range: 40–200, level: high image quality. The mean DLP was 127, and in no instance did it exceed 157.

The acquired images were classified according to quality, and only those 14 patient studies (28 condyles) allowing proper image registration were included in the analysis. Examinations showing significant misalignment or other registration errors between the SPECT and CT images were excluded.

### Synthetic CT

With the assistance of the AI Group at Mediso Ltd., SyCT images were generated directly from patients’ raw SPECT data through a multi-stage processing pipeline implemented and executed within Mediso’s released and approved software (Mediso InterView XP, 3.9). In this paper, we outline the internal processing steps as realized at Mediso, reflecting our close technical collaboration; however, the underlying implementation is a Mediso-developed, authorized product solution. The aim of this publication is not to provide an exhaustive technical description or methodological analysis of the software, but to validate its performance and assess its real-world use on clinical data.

The pipeline comprises noise reduction, a non–attenuation-corrected (non-AC) reconstruction integrated into a neural-network framework, and a final SPECT-to-CT conversion. Noise suppression is performed using a 3D U-Net–based denoiser trained following the approach of Kovács et al. [14, 15], modified to process full 3D SPECT projections instead of 2D planar bone scintigraphy. To achieve this, 3D convolutional and pooling operators are used in place of their 2D counterparts, enabling volumetric feature extraction from the entire SPECT volume.

The SyCT generator employs a deep U-Net architecture composed of six encoder–decoder stages, each containing three convolutional layers. The number of feature channels increases progressively with depth, beginning with four filters in the first block and doubling at each subsequent level until reaching 512 filters in the deepest layer. In total, the network comprises approximately 28.3 million trainable parameters and produces its final output through a linear convolutional layer with identity activation.

To ensure that the networks learned only physiologically valid structures, rigorous data cleaning precedes training. Manual selection and cleaning were performed by medical experts and application specialists participating in the Mediso development team, following a standardized protocol specifying the parameters and artifacts to be evaluated. During this process, experts not only excluded low-quality data but also assigned comprehensive metadata to each scan through DICOM information extraction (e.g., patient gender, weight, height, radiopharmaceutical dose, number of acquired field-of-views, acquisition time per FOV, total counts) and manual labels (e.g., complete body presence, registration accuracy, artifact type and presence, availability of extended or MAR reconstructions, signs of pathological enhancement, and the number of lesions detected). This procedure ensures consistent and high-quality training inputs while promoting a thorough understanding of the dataset.

From an initial dataset of 1350 paired SPECT/CT scans obtained from Scanomed, 518 high-quality pairs were retained after manual inspection by the AI Developers and collaborating medical specialists. A predominance of data was available for the chest (*n* = 512), abdomen (*n* = 239), and pelvic (*n* = 450) regions, while the number of examinations for the skull (*n* = 92) and lower extremities (*n* = 57) fell into a lower range. Exclusion criteria include SPECT–CT misregistration greater than 2 mm, transaxial body truncation, abnormal noise accumulation, visible artifacts in either modality (e.g., urinary activity, paravasate, metal streaks), activity outside the patient’s body, or any form of motion artifact. All models are implemented and trained using the PyTorch framework.

Importantly, to prevent any possibility of data leakage, the 14 UCH cases used for evaluation were entirely outside the development dataset: they were not accessed or used at any stage of product development (including training, tuning, or internal testing) and therefore constitute a fully independent validation cohort.

### SPECT reconstruction

SPECT reconstructions were then performed using both the original CT and the SyCT, applying identical parameters in Interview XP version 3.09 (Mediso Ltd., Budapest, Hungary). The reconstruction parameters were as follows: Tera-Tomo 3D SPECT reconstruction algorithm; bone regularization; matrix size, 128 × 128; 48 iterations; 4 subsets; CT-based attenuation correction; scatter correction enabled; and the quantitative reconstruction module activated. This module enables quantitative SPECT by normalizing reconstructed voxel values to activity concentration (Bq/mL) through incorporation of system sensitivity modeling (via Monte Carlo simulation) and an activity calibration factor derived from a prior measurement with known total activity. This approach has been validated for quantitative accuracy in performance evaluations of similar SPECT systems [16–19].

The SPECT images reconstructed with the original CT were designated as CTAC (CT Attenuation Correction), while those reconstructed using the AI-generated SyCT were designated as SyCTAC (Synthetic CT Attenuation Correction).

### Evaluation

The image processing was performed using the Interview Fusion 3.10.009.0000 reporting and evaluation software (Mediso Ltd., Budapest, Hungary). The software interface was divided into two equal parts, allowing simultaneous display of the CTAC-SPECT/CT and SyCTAC-SPECT/SyCT images. Since both reconstructions were derived from the same SPECT acquisition, they were in perfect spatial alignment. The cursors were linked across the two displays, ensuring that navigation in one image always corresponded to the identical slice in the other.

During the evaluation, the right and left mandibular condylar regions were identified on the CT images. Correspondingly, spherical VOIs (volume of interest) with a diameter of 2 cm were placed over both condyles on the CTAC images. Using the same approach, a 2 cm spherical VOI was also placed over the clivus region. Subsequently, the condylar and clivus VOIs were copied to the SyCTAC images in the identical positions. Of the 4.2 mm voxels forming the images, only those with more than 50% of their volume in the spherical VOI were included. Using the > 50% volume inclusion rule on 4.2 mm voxels, the mean number of voxels per VOI was 56.4 ± 2.0 (SD; range 52–62 across the 42 VOIs), ensuring adequate sampling for robust variance estimation of SUVmax and SUVmean. Each condyle’s uptake has to normalized independently to the clivus because of better sensitivity, specificity and significantly better method compared to relative uptake method, most in bilateral condylar hyperplasia patients [5].

Based on the VOIs placed over the mandibular condyles and the clivus, SUVmax and SUVmean values were determined on both the CTAC and SyCTAC images and two kinds of uptake ratios were calculated:

#### Relative uptake fractions

- **rUmax**,_**T**_: Relative maximum uptake of the unilateral condyle normalized to the summed max uptake of both condyles

1 $$r{Umax}_{T}=\frac{{SUVmax}_{\text{unilateral condyle}}}{{SUVmax}_{\text{right condyle}}+{SUVmax}_{\text{left condyle}}}$$

- **rUmean**,_**T**_: Relative mean uptake of the unilateral condyle normalized to the summed mean uptake of both condyles

2 $$r{Umean}_{T}=\frac{S{UVmean}_{\text{unilateral condyle}}}{{SUVmean}_{\text{right condyle}}+{SUVmean}_{\text{left condyle}}}$$

Because the differences of the uptake fractions obtained by the two AC-correction methods on the two sides are additive inverses of each other, we analyzed only one value per patient: the value from the „affected” side, that had a higher mean uptake in the CT-corrected image.

#### Relative uptake normalized to the clivus as reference area

- **rUmax**,_**C**_: Relative maximum uptake of the unilateral condyle divided by the max clivus uptake

3 $$r{Umax}_{C}=\frac{S{UVmax}_{\text{unilateral condyle}}}{{SUVmax}_{\mathrm{clivus}}}$$

- **rUmean**,_**C**_: Relative mean uptake of the unilateral condyle divided by the mean clivus uptake

4 $$r{Umean}_{C}=\frac{{SUVmean}_{\mathrm{unilateralcondyle}}}{{SUVmean}_{\mathrm{clivus}}}$$

Based on the hypothesis that attenuation correction artifacts from SPECT-based maps can occur unilaterally, independent of the contralateral side, we included in the analysis the uptake values of both condyles normalized to the reference-area separately. This solution allows us to capture the side-specific variability and provides a more comprehensive assessment of the method’s performance across all clinical scenarios, rather than limiting the analysis to a single ‘affected’ side.

### Statistical analysis

Statistical analyses were performed using SPSS version 31 (IBM Corp., Armonk, NY, USA). Normality was assessed with the Shapiro-Wilk test. Agreement between the two quantitative approaches (CTAC-based vs. SyCTAC-based ratios) was evaluated differently for the two groups of uptake indices.

Bland–Altman analysis was applied to the fractional uptakes of the “affected” side.

For the uptake values normalized to a reference area, the measurements from the two sides are not statistically independent. To assess agreement between the two methods, we used a mixed-effects model [20] with ‘patient’ as a random effect, thereby accounting for the correlation between the two condyles belonging to the same individual. This approach allows us to estimate agreement using all data points without violating the assumption of independence. From this model, we derived the confidence interval of the differences and the limits of agreement, with standard errors correctly adjusted for clustering.

In the Bland–Altman plots, the intervals calculated by the linear mixed-effects model are shown. Differences were also visualized using box-and-whisker plots. A *p-value* < 0.05 was considered statistically significant.

## Results

### Visual evaluation

**Fig. 1:**
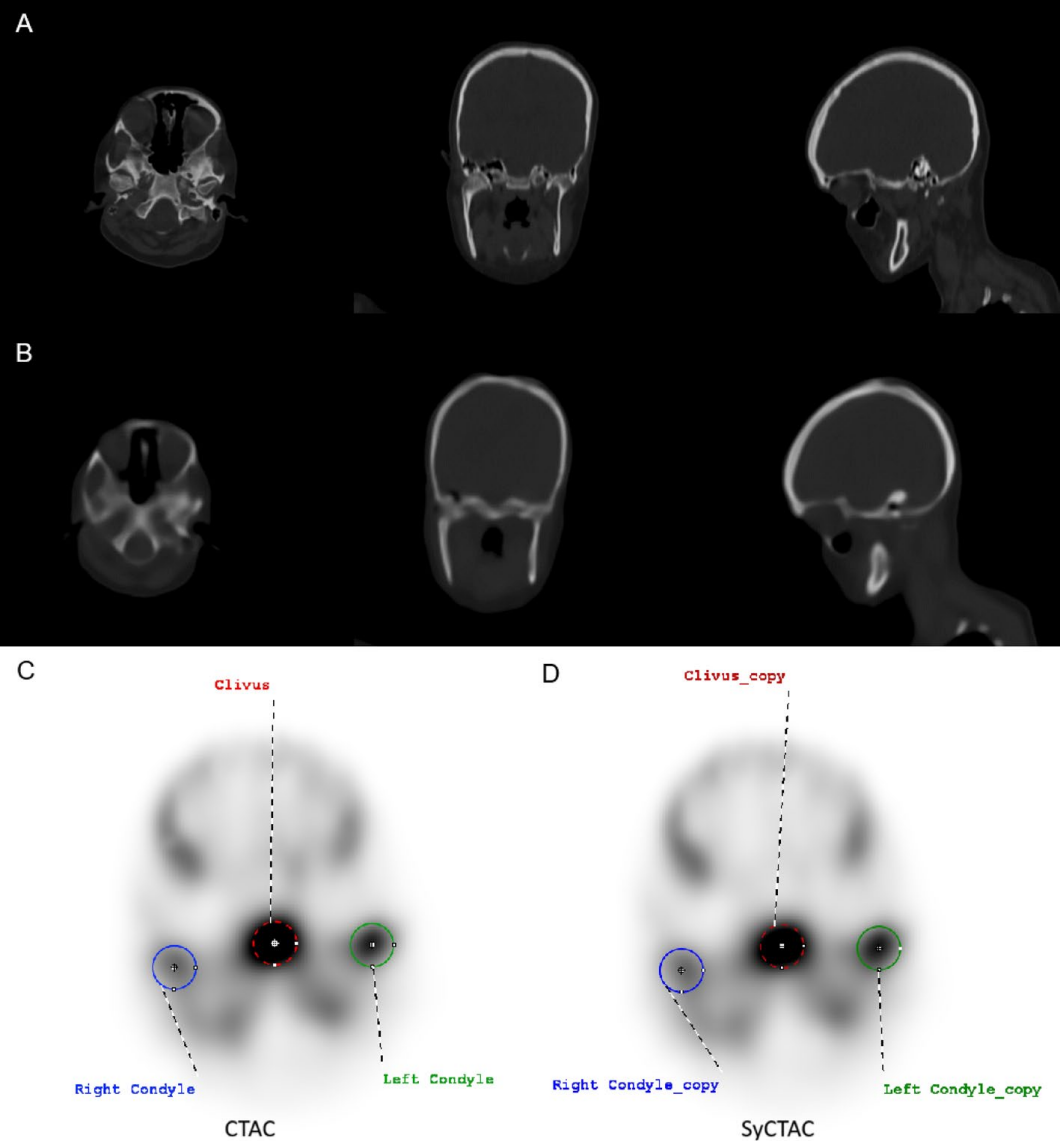
Axial, coronal and sagittal slices of CT (**A**) and SyCT (**B**) images. Axial slices of the CTAC (**C**) and SyCTAC (**D**) images with spherical VOIs (diameter: 2 cm) placed over both condyles and the clivus

Between 2014 and 2024, a total of 14 patients with UCH underwent ^99m^Tc-MDP SPECT/CT examinations at our institution that met the inclusion criteria. Visual evaluations were independently performed by three nuclear medicine specialists, who reported no substantial differences between the SPECT images reconstructed with CT and those reconstructed with SyCT. Specifically, no differences were noted regarding image intensity display, lesion detectability, or the feasibility of VOI placement (Fig. 1).

### Quantitative evaluation

Clinical data of the patients, examination parameters, and relative uptake fractions are presented in Table 1. According to the literature, a condylar relative uptake of 55% or greater was considered indicative of active UCH [1, 4, 5]. Using this threshold, active UCH was identified in 8 of the 14 patients based on rUmax,_T_, and no difference in diagnostic outcome was observed between the rUmax,_T_ values calculated from CTAC and SyCTAC images. For rUmean,_T_, the diagnostic outcome differed in two patients between the CTAC- and SyCTAC-derived values.

**Table 1:** Clinical data of the patients, examination parameters, and relative uptake fractions

ID	Age (year)	Sex	Injected activity (MBq)	Year of examination	Scan quality notes	rUmax,_T_ CTAC (%)	rUmax,_T_ SyCT (%)	rUmean,_T_ CTAC (%)	rUmean,_T_ SyCT (%)
1	41.21	f	571	2015	Optimal	50.2	48.9	58.8	55.4
2	20.12	m	572	2014	Optimal	53.2	51.7	58.9	58.2
3	16.29	f	437	2016	Optimal	49.0	48.1	51.9	51.0
4	29.03	f	589	2016	Optimal	54.2	53.2	50.0	48.8
5	32.46	f	578	2017	Optimal	51.4	51.4	52.2	52.7
6	17.97	f	486	2017	Optimal	56.5	55.8	62.8	55.7
7	20.53	f	582	2018	Optimal	76.8	77.4	72.0	72.7
8	19.81	f	537	2019	Optimal	67.7	67.5	60.1	59.2
9	16.96	f	444	2019	Optimal	65.1	63.1	62.1	63.0
10	42.29	f	562	2019	Optimal	54.9	52.2	55.8*	52.1*
11	20.40	f	582	2024	Optimal	64.1	64.8	59.8	61.1
12	22.04	f	562	2024	Optimal	59.4	58.2	57.9	57.1
13	21.13	f	583	2024	Optimal	59.2	59.9	55.4*	54.0*
14	19.81	m	540	2019	Optimal	87.2	86.4	82.9	82.4

*Using the 55% threshold value, the diagnostic outcome differed in two patients between the CTAC- and SyCTAC-derived rUmean,_T_ values

### Statistical analysis

#### Tests of normality

According to the Shapiro-Wilk tests, the distributions of the calculated relative uptake ratios derived from CTAC and SyCTAC images did not differ significantly from a normal distribution.

#### Bland–Altman analysis of the relative uptake fractions

Agreement between the relative uptake ratios obtained from CTAC and SyCTAC was evaluated using Bland–Altman analysis. The Bland–Altman plots display the signed differences of the relative maximum and mean uptake ratios against the averages of the ratios derived from the two methods.

**Fig. 2:**
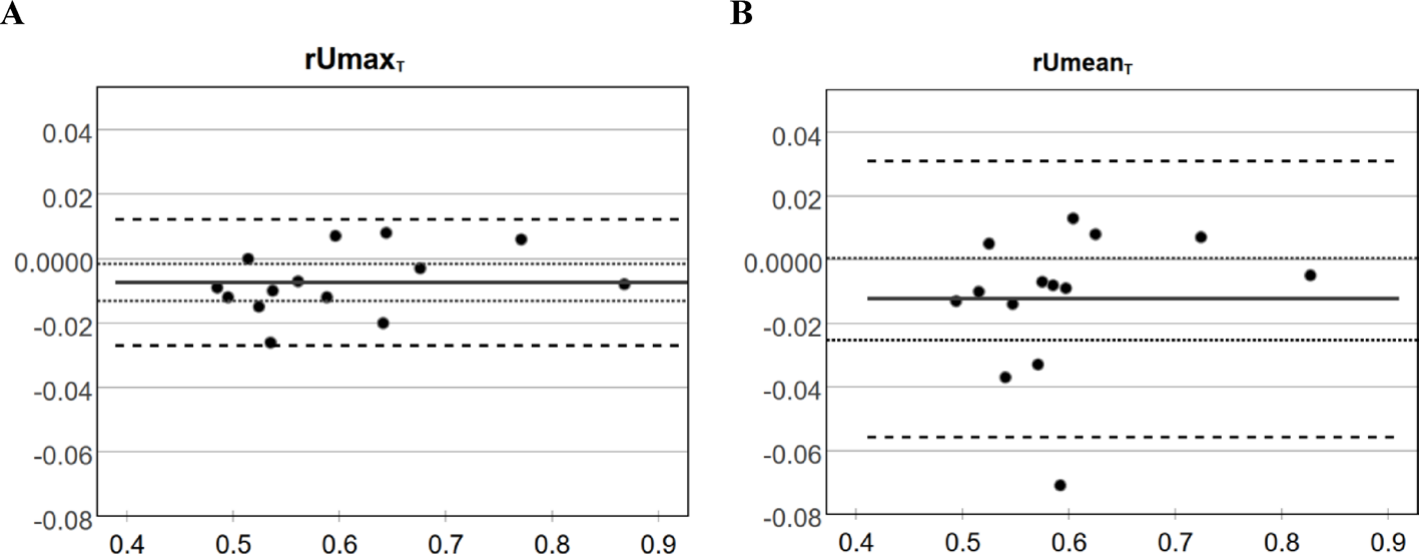
Bland–Altman analysis of relative uptake ratios derived from CTAC and SyCTAC images. Plots illustrating the signed differences of *rUmax*,_*T*_ (**A**) and *rUmean*,_*T*_ (**B**) against their corresponding averages. Continuous line: mean difference; dotted lines: confidence interval of the difference; dashed lines: limits of agreement

As shown in Fig. 2, with SyCTAC there was a slight underestimation of the relative uptake fractions; for rUmax,_T_, *p* < 0.05, while for rUmean,_T_ the scatter of the differences was greater, resulting in *p* > 0.05. The limits of agreement show that the differences are clinically not significant.

#### Analysis of the relative uptake normalized to the clivus

**Fig. 3:**
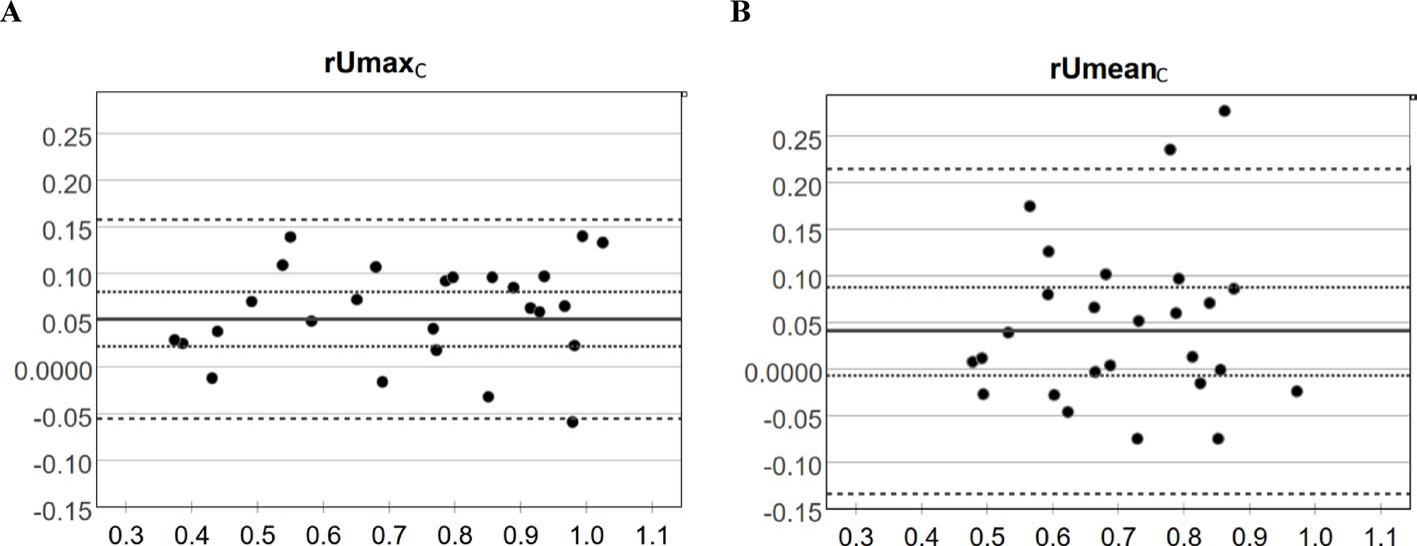
Bland–Altman analysis of relative uptake ratios derived from CTAC and SyCTAC images. Plots illustrating the signed differences of *rUmax*,_*C*_ (**A**) and *rUmean*,_*C*_ (**B**) against their corresponding averages. Continuous line: mean difference; dotted lines: confidence interval of the difference; dashed lines: limits of agreement

The results for the relative uptake ratios normalized to the clivus are presented in the form of Bland–Altman plots, where the confidence intervals and the limits of agreement were derived by applying linear mixed models (Fig. 3). It revealed systematic bias between the CTAC- and SyCTAC-based measurements for rUmax,_C_ (*p* = 0.002; mean difference = 0.051), while for rUmean,_C_ the difference (mean = 0.041) was not significant (*p* > 0.05) due to a higher scatter of differences. One dataset was excluded from the clivus-normalized Bland–Altman analysis owing to a suspected reconstruction scaling error that produced unrealistically high absolute SUV values (while preserving correct relative ratios). This case was retained in all other analyses.

### Differences between SyCTAC- and CTAC-based relative uptake values

**Fig. 4:**
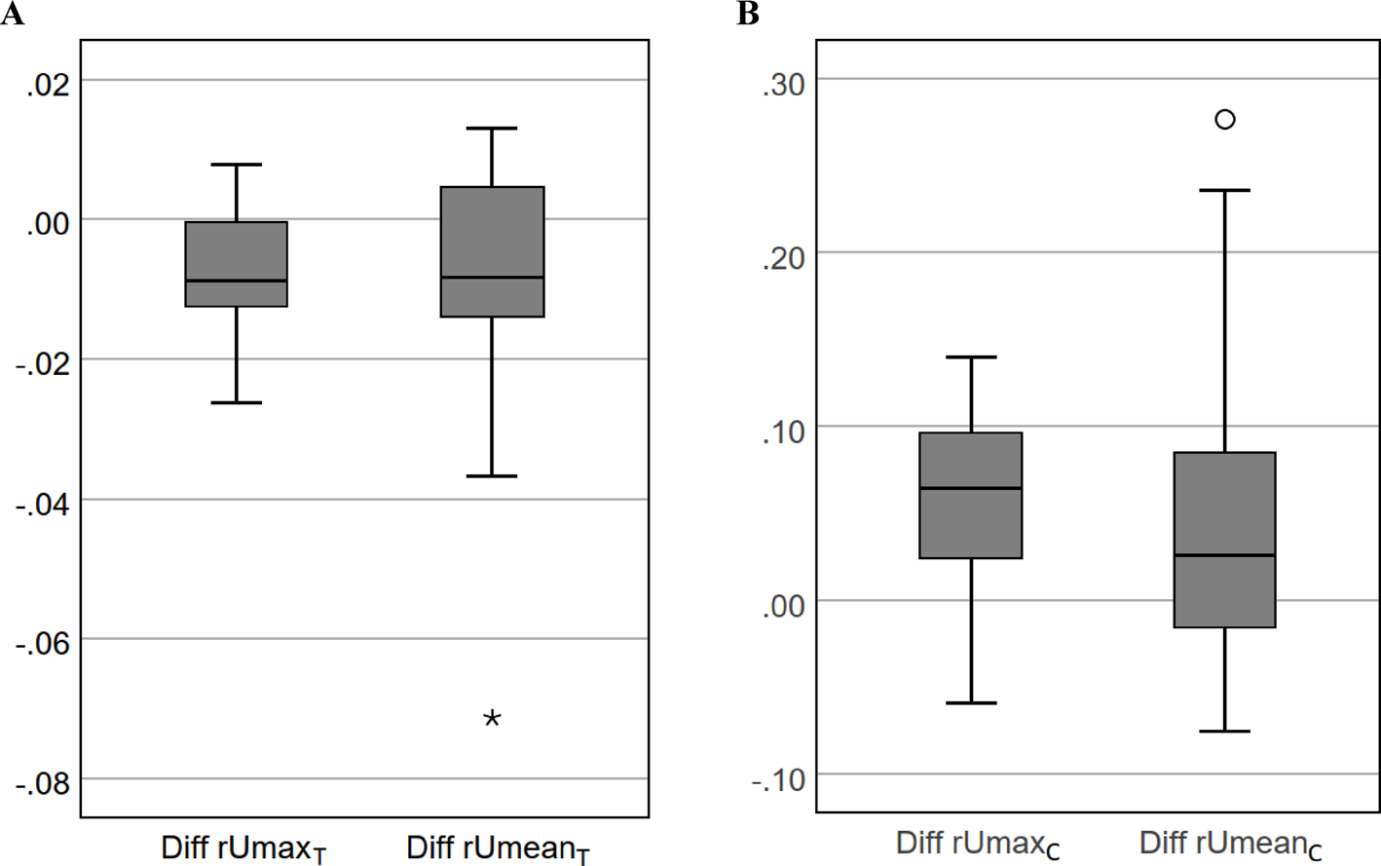
Box-and-whisker plots illustrating the differences between SyCTAC- and CTAC-based relative uptake ratios. **A** Differences in rUmax,_T_ and rUmean,_T_, representing the relative maximum and mean uptake of the “affected” condyle normalized to the summed uptake of both condyles. **B** Differences in rUmax,_C_ and rUmean,_C_, representing the relative maximum and mean condylar uptake normalized to the uptake of the clivus

The differences in the relative uptake ratios between CTAC and SyCTAC, presented in Bland–Altman plots in Figs. 2 and 3, were also illustrated using box-and-whisker plots in Fig. 4. While SyCTAC slightly underestimated the relative uptake fractions (see Fig. 4A), it slightly overestimated the values relative to the deeper‑lying clivus. Variability was lower for the max than for the mean values.

## Discussion

Recent advances in artificial intelligence have enabled the generation of µ-map or SyCT directly from nuclear medicine emission data. This approach has been proposed as a way to preserve quantitative accuracy while potentially reducing or eliminating the need for a true CT scan [11, 21], thereby aligning with ALARA-based recommendations [10].

This study examined whether AI-generated SyCT images can provide attenuation maps suitable for quantitative ^99m^Tc-MDP bone SPECT reconstruction in patients with UCH. The visual assessment performed by three nuclear medicine specialists confirmed that SyCTAC-based reconstructions did not differ perceptibly from CTAC in overall image quality, lesion visibility, or suitability for VOI placement.

Across the evaluated parameters, SyCTAC demonstrated close agreement with CTAC in the quantification of condylar uptake, when uptake values were normalized to the summed activity of both condyles, although tended to slightly underestimate the update fraction of the affected side. These findings indicate that SyCT-derived attenuation correction can reasonably estimate the relative differences in uptake between the right and left condyles and maintain the contrast relationships needed for diagnostic assessment. This is of particular relevance in UCH, where clinical decision-making often relies on the degree of asymmetry rather than absolute uptake values [1]. Bland–Altman analysis confirmed no evidence of clinically significant bias or proportional error, highlighting the robustness of SyCTAC for condyle-to-condyle comparisons.

Greater variability was observed in ratios normalized to the clivus, with statistically significant differences between SyCTAC and CTAC. These discrepancies may relate to the anatomical and functional characteristics of the clivus, a dense osseous structure with relatively low metabolic activity, making SUV estimation more sensitive to small deviations in attenuation mapping. AI-based SyCT generation may also be less accurate in regions where tracer uptake provides limited structural information. In addition, the complex bone-air interfaces at the skull base and the limited local emission signal may challenge emission-driven SyCT generation, causing minor but systematic attenuation-map differences that become amplified when a single reference VOI is used for normalization. Nonetheless, the significant correlations observed for both maximum and mean clivus-normalized ratios indicate that SyCTAC still preserved the overall ranking of uptake values. Importantly, clinical assessment of UCH most commonly relies primarily on condyle-to-condyle comparisons rather than reference-region normalization, reducing the practical impact of these differences [1].

These AI-driven approaches have been particularly effective in myocardial perfusion SPECT [7, 11, 12], quantitative thyroid SPECT imaging [22] and kidney SPECT imaging for glomerular filtration rate (GFR) measurement [12], with the added benefit of reducing patient radiation exposure. Apostolopoulos et al. reported that in ^99m^Tc myocardial perfusion imaging among existing deep learning AC methods, attention-enhanced U-Nets and conditional GANs showed the most promising performance, particularly when validated against independent datasets [7]. Miyai et al. proposed a deep learning-based AC method to generate pseudo-CT images using non-AC SPECT images in ^99m^Tc-labeled galactosyl human serum albumin (GSA) hepatic SPECT/CT imaging, demonstrating that the resulting AC accuracy was comparable to conventional CTAC using real CT image [11].

SyCTAC may offer several advantages over conventional CTAC for UCH imaging and beyond. Eliminating the need for CT acquisition reduces radiation exposure [9], which is particularly relevant for the typically young patient population undergoing evaluation for UCH. Although SyCT images inherently exhibit lower spatial resolution than diagnostic CT scans—as expected, given their generation from non-attenuation-corrected SPECT emission data via deep learning—this does not adversely impact the accuracy of attenuation correction in quantitative SPECT imaging. In practice, attenuation maps (µ-maps) derived from either CT or SyCT are resampled and potentially smoothed to align with the lower-resolution SPECT voxel grid during reconstruction, ensuring appropriate handling of tissue boundaries and minimizing partial volume effects. This process mitigates any concerns arising from the smoother transitions in SyCT, as demonstrated by the excellent agreement in relative condylar uptake ratios and lack of significant bias in our results (except for a small positive bias in clivus-normalized ratios, which is clinically negligible). Prior studies in myocardial perfusion SPECT have similarly shown that deep learning-generated synthetic attenuation maps provide quantitative accuracy comparable to CTAC, without resolution-related degradation at boundaries [23–25]. AI-generated attenuation maps also eliminate the risk of misregistration between SPECT and CT volumes that can compromise quantitative accuracy. From a workflow standpoint, SyCTAC facilitates retrospective analyses even when CT data are missing or unusable. Moreover, AI-based AC could potentially enable quantitative SPECT imaging in settings where hybrid SPECT/CT systems are unavailable, broadening access to advanced diagnostic capabilities.

This study has limitations. The cohort size was modest due to the rarity of UCH and the strict inclusion criteria imposed to ensure high-quality image registration. Larger, multicenter studies are needed to confirm the generalizability of these findings across different scanners, acquisition protocols, and patient populations. Additionally, the present work focused primarily on relative uptake ratios rather than absolute SUVs; future investigations should explore the behavior of SyCTAC in absolute quantification, which may be relevant for other clinical applications.

A further limitation is that our quantitative evaluation was restricted to VOIs in the mandibular condyles and clivus, which are predominantly bony regions and do not specifically assess challenging inter-tissue edge regions, such as bone-air (e.g., sinuses) or bone-soft tissue interfaces. These boundaries are known to pose potential difficulties for emission-driven SyCT methods, where misclassification or smoothing could theoretically introduce minor attenuation artifacts [26, 27]. While our results showed excellent agreement in relative uptake ratios without apparent boundary-related biases, future prospective studies should incorporate targeted analyses at such edges to confirm the technical robustness of SyCTAC in craniofacial applications.

If SyCTAC is implemented, mixing CTAC- and SyCTAC-derived quantitative metrics within the same patient or across retrospective cohorts may introduce systematic shifts due to differences in attenuation correction. This issue may be particularly relevant when reference-region normalization is applied, as small systematic differences in the reference region can influence the normalized uptake values. Our results support this possibility, as a small bias was observed when clivus normalization was used. Consequently, consistent use of the same attenuation correction approach should be considered when performing quantitative analyses.

## Conclusions

In conclusion, this study demonstrates that SyCTAC provides quantitative results comparable to CTAC for assessing condylar metabolic asymmetry in patients with UCH. These findings highlight the potential of AI-based AC to serve as a low-radiation, CT-independent alternative to conventional SPECT/CT, supporting more flexible and accessible quantitative imaging strategies. As AI methodologies continue to mature, SyCTAC may become an increasingly valuable tool in nuclear medicine.

## Data Availability

The datasets generated during and/or analyzed during the current study are available from the corresponding author on reasonable request.
